# Predicting coronary artery calcified plaques using perivascular fat CT radiomics features and clinical risk factors

**DOI:** 10.1186/s12880-022-00858-7

**Published:** 2022-07-29

**Authors:** Guo-qing Hu, Ya-qiong Ge, Xiao-kun Hu, Wei Wei

**Affiliations:** 1 Department of Radiology, The First Affiliated Hospital of USTC, Wannan Medical College, Wuhu, 241002 Anhui China; 2 GE Healthcare China, No. 1 Huatuo Road, Pudong New Town, Shanghai, 210000 China; 3 Department of Radiology, The First Affiliated Hospital of USTC, Division of Life Sciences and Medicine, University of Science and Technology of China, Hefei, 230001 Anhui China

**Keywords:** Coronary artery, Plaque, Radiomics, Perivascular fat attenuation

## Abstract

**Objective:**

The purpose of this study was to develop a combined radiomics model to predict coronary plaque texture using perivascular fat CT radiomics features combined with clinical risk factors.

**Methods:**

The data of 200 patients with coronary plaques were retrospectively analyzed and randomly divided into a training group and a validation group at a ratio of 7:3. In the training group, The best feature set was selected by using the maximum correlation minimum redundancy method and the least absolute shrinkage and selection operator. Radiomics models were built based on different machine learning algorithms. The clinical risk factors were then screened using univariate logistic regression analysis. and finally a combined radiomics model was developed using multivariate logistic regression analysis to combine the best performing radiomics model with clinical risk factors and validated in the validation group. The efficacy of the model was assessed by a receiver operating characteristic curve, the consistency of the nomogram was assessed using calibration curves, and the clinical usefulness of the nomogram was assessed using decision curve analysis.

**Results:**

Twelve radiomics features were used by different machine learning algorithms to construct the radiomics model. Finally, the random forest algorithm built the best radiomics model in terms of efficacy, and this was combined with age to construct a combined radiomics model. The area under curve for the training and validation group were 0.98 (95% confidence interval, 0.95–1.00) and 0.97 (95% confidence interval, 0.92–1.00) with sensitivities of 0.92 and 0.86 and specificities of 0.99 and 1, respectively. The calibration curve demonstrated that the nomogram had good consistency, and the decision curve analysis demonstrated that the nomogram had high clinical utility.

**Conclusions:**

The combined radiomics model established based on CT radiomics features and clinical risk factors has high value in predicting coronary artery calcified plaque and can provide a reference for clinical decision-making.

## Background

Coronary artery disease is a common cardiovascular disease that seriously endangers human health, and the most important pathological basis for the occurrence of this disease is atherosclerosis. Research has shown that the coronary atherosclerotic plaque composition is closely associated with obstructive coronary stenosis and acute coronary syndromes [1, 2]. Calcified plaques contain mainly fibrous tissue hyperplasia and calcified material with a hard texture and are relatively stable. In contrast, non-calcified plaques contain mainly lipid, old hemorrhage, and inflammatory cells that rupture, dislodge, or induce thrombosis because of instability, leading to the development of coronary artery disease [3]. Petretta et al. [4] demonstrated that the number of non-calcified plaques was an independent risk factor for the development of cardiovascular disease. Therefore, effective detection of plaque composition is of great significance for the clinical management and prognosis of patients. Coronary CT angiography (CCTA) has high-density resolution and is effective in detecting coronary plaques and facilitating judgments about the constituent components of plaques, but with limited accuracy [5, 6]. In recent years, perivascular fat has received increased attention from researchers. Perivascular adipose tissue (PVAT) is the adipose tissue that surrounds the outer membrane of blood vessels and is closely associated with the development of atherosclerosis, the degree of coronary stenosis, plaque stability, and prognosis [7–13]. Radiomics, an emerging post-processing method, can be used for disease prediction and early diagnosis by extracting and quantifying useful features in medical imaging data [14, 15]. In this study, we analyzed and modeled the CT radiomics features of perivascular fat and the clinical risk factors of patients to investigate the value of the model in predicting the nature of coronary plaque.

## Methods

### Patients’ information

We retrospectively analyzed images of 200 patients who underwent CCTA at our hospital from June 2019 to May 2020 and were diagnosed with calcified or non-calcified plaques. Of these patients, 105 had calcified plaques (calcified volume of > 70%) and 95 had non-calcified plaques (calcified volume of < 30%).When a patient’s image had multiple plaque lesions, the lesion with the clearest imaging features was selected for analysis. The inclusion criteria of this study were (1) the acquisition of complete CT-enhanced images strictly according to a fixed scan, (2) no history of coronary artery disease (no previous myocardial infarction or intervention), and (3) a > 50-mm length of coronary artery where the plaque was located. The exclusion criteria were (1) significant image noise or other quality problems, (2) both non-calcified and calcified plaques in a single coronary artery, and (3) absence of clinical symptoms. Data regarding age, sex, diseased blood vessels, hypertension, diabetes, and hyperlipidemia were collected from the medical records.

### Image acquisition

Each patient underwent cardiac coronary plain and enhanced CT scans (Discovery CT750 or GE Optima CT680; GE Healthcare, Chicago, IL, USA). A total dose of 84 mL of iohexol contrast agent with 60 mL of saline was injected into the elbow vein through a high-pressure syringe at an injection rate of 4.5 mL/s. The acquisition parameters were as follows: tube voltage, 120 KVp; tube current, 450–480 mA; layer thickness, 0.625 mm; and layer spacing, 0.625 mm. Images were acquired in Digital Imaging and Communications in Medicine format from the workstation.

### Medical image segmentation and feature extraction

The perivascular fat was outlined by ITK-SNAP (www.itksnap.org) at 10–50 mm proximal to the coronary artery, and the voxels with CT values between − 190 and − 30 HU over the same radial distance as the vessel diameter were defined as PVAT [7, 16]. Feature extraction was further performed by Z-score normalization of the original image as well as determination of the region of interest by Artificial Intelligence Kit software (A.K. Software, version 3.3.0.R; GE Healthcare). The feature types included the first order, shape, gray-level co-occurrence matrix (GLCM), gray-level size zone matrix (GLSZM), and gray-level run-length matrix (GLRLM), as well as log and wavelet transformed features. The extracted features comprised 198 first order features, 14 shape features, 264 GLCM features, 176 GLSZM features, and 176 GLRLM features. The specific feature classification is shown in Table 1.

**Table 1:** All extracted features and classifications

	Original	Log-sigma		Wavelet							
		2.0	3.0	HHH	HHL	HLH	HLL	LHH	LHL	LLH	LLL
Shape	14	0	0	0	0	0	0	0	0	0	0
First Order	18	18	18	18	18	18	18	18	18	18	18
GLCM	24	24	24	24	24	24	24	24	24	24	24
GLSZM	16	16	16	16	16	16	16	16	16	16	16
GLRLM	16	16	16	16	16	16	16	16	16	16	16

### Feature selection and model building

**Fig. 1:**
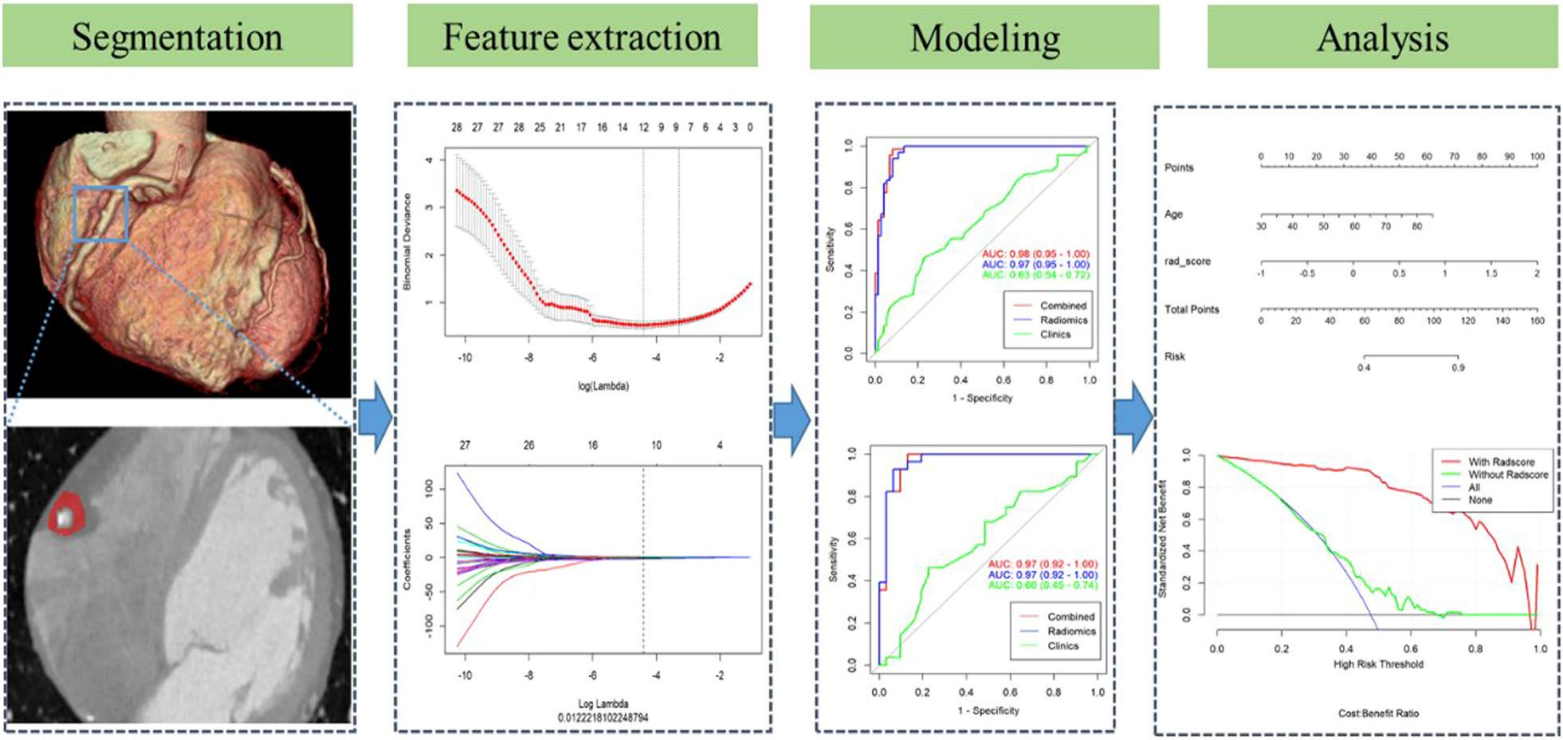
Workflow of the radiomics analysis

The data were divided into a training group and validation group at a ratio of 7:3. Considering the imbalance of the data, Synthetic Minority Oversampling Technique (SMOTE) was used to oversample the minority class to get a balanced data distribution in the training set, then the features were first screened in the training group using the maximum correlation minimum redundancy (mRMR) method. They were then further screened using the least absolute shrinkage and selection operator (LASSO) tenfold cross-validation method to find the lambda value when the minimum binomial deviation was 0, and the features with coefficients that were not 0 at this point were retained (Fig. 1). The final retained features were analyzed using different machine learning algorithms (glm, K-nearest neighbor, support vector machine with radial basis function kernel [svmRadial], random forest, and neural network) to build the radiomics model. The stability and predictive performance of each model over tenfold cross-validations repeated with 10 times were analyzed using relative standard deviations (RSD%) which is defined as:

$${\text{RSD}}\% =\upsigma \;{\text{AUC}}/\upmu \;{\text{AUC}} \times 100\% ,$$

where σ AUC is the standard deviation of the 100 AUC values and µ AUC is the mean of the 100 AUC values, then the models were validated in the validation group, and the best performing radiomics model was finally combined with clinical risk factors using multivariate logistic regression analysis to construct a combined radiomics model. The utility of the models was analyzed using decision curves with the Hosmer–Lemeshow test [17, 18] to create calibration curves to identify agreement between the probabilities predicted by column line plots and the actual rates of calcified plaque in the training and validation group.

### Statistical analysis

All statistical analyses were performed using R software version 3.5.0 (http://www.r-project.org), and *p* values of < 0.05 were considered statistically significant. For continuous variables such as age, the Kolmogorov–Smirnov method was used to test whether the measures conformed to a normal distribution, using the independent-samples t-test for a normal distribution and the Mann–Whitney U test for non-normality. For categorical variables, the chi-square test or Fisher’s exact test was used. Univariate and multivariate logistic regression analysis was used to select the most discriminating risk factors and build the combined radiomics models. The efficacy of the model was assessed using an ROC curve, the consistency of the model was assessed using calibration curves, and the usefulness of the model was assessed using clinical decision analysis.

## Results

### Clinical characteristics

In total, 200 patients were included in this study; 105 (52.5%) had calcified plaques, and 95 (47.5%) had non-calcified plaques. The patients’ basic clinicopathological characteristics were compared between the calcified and non-calcified plaque groups in the training and validation group are shown in Table 2. Only age was statistically different in the training group (*p* < 0.05), all other clinical factors were not statistically different (*p* > 0.05).

**Table 2:** Comparison of patient and clinical risk factors in training and validation group

	Training group (n = 141)			Validation group (n = 59)		
	Calcified plaque group (n = 74)	Non-calcified plaque group (n = 67)	*p*	Calcified plaque group (n = 31)	Non-calcified plaque group (n = 28)	*p*
Age (years)	61.95 ± 10.15	57.76 ± 10.04	0.007	63.58 ± 9.02	59.93 ± 9.92	0.212
Gender			0.234			0.470
Male	38 (51.4%)	46 (68.7%)		18 (58.1%)	14 (50.0%)	
Female	36 (48.6%)	21 (31.3%)		13 (41.9%)	14 (50.0%)	
Hypertension			0.538			0.090
Yes	32 (43.2%)	37 (55.2%)		18 (58.1%)	18 (64.3%)	
No	42 (56.8%)	30 (44.8%)		13 (41.9%)	10 (35.7%)	
Diabetes			0.229			0.597
Yes	10 (13.5%)	6(9.0%)		6 (19.4%)	5 (17.9%)	
No	64 (84.5%)	61(91.0%)		25(80.6%)	23 (82.1%)	
Hyperlipidemia			0.248			0.066
Yes	5 (6.8%)	6 (9.0%)		5 (16.1%)	2 (7.1%)	
No	69 (93.2%)	61 (91.0%)		26 (83.9%)	26 (92.9%)	
Diseased blood vessel			0.808			0.226
LM	4 (5.4%)	4 (6.0%)		3 (9.7%)	1 (3.5%)	
LAD	43 (58.1%)	40 (59.7%)		18 (58.1%)	16 (57.1%)	
D	1 (1.4%)	3 (4.5%)		0 (0)	2 (7.1%)	
LCx	10 (13.5%)	4 (6.0%)		3 (9.7%)	1 (3.6%)	
RCA	14 (18.9%)	15 (22.3%)		7 (22.5%)	8 (28.6%)	
PDA	2 (2.7%)	1 (1.5%)		0 (0)	0 (0)	

*LM* left main artery, *LAD* left anterior descending artery, *D* diagonals, *LCx* left circumflex artery, *RCA* right coronary artery, *PDA* posterior descending artery

Qualitative variables are expressed as n (%) and were analyzed using the chi-square test or Fisher’s exact test, and quantitative variables are expressed as mean ± standard deviation and were analyzed using the t-test. A *p* value of < 0.05 was considered statistically significant

### Feature extraction

**Fig. 2:**
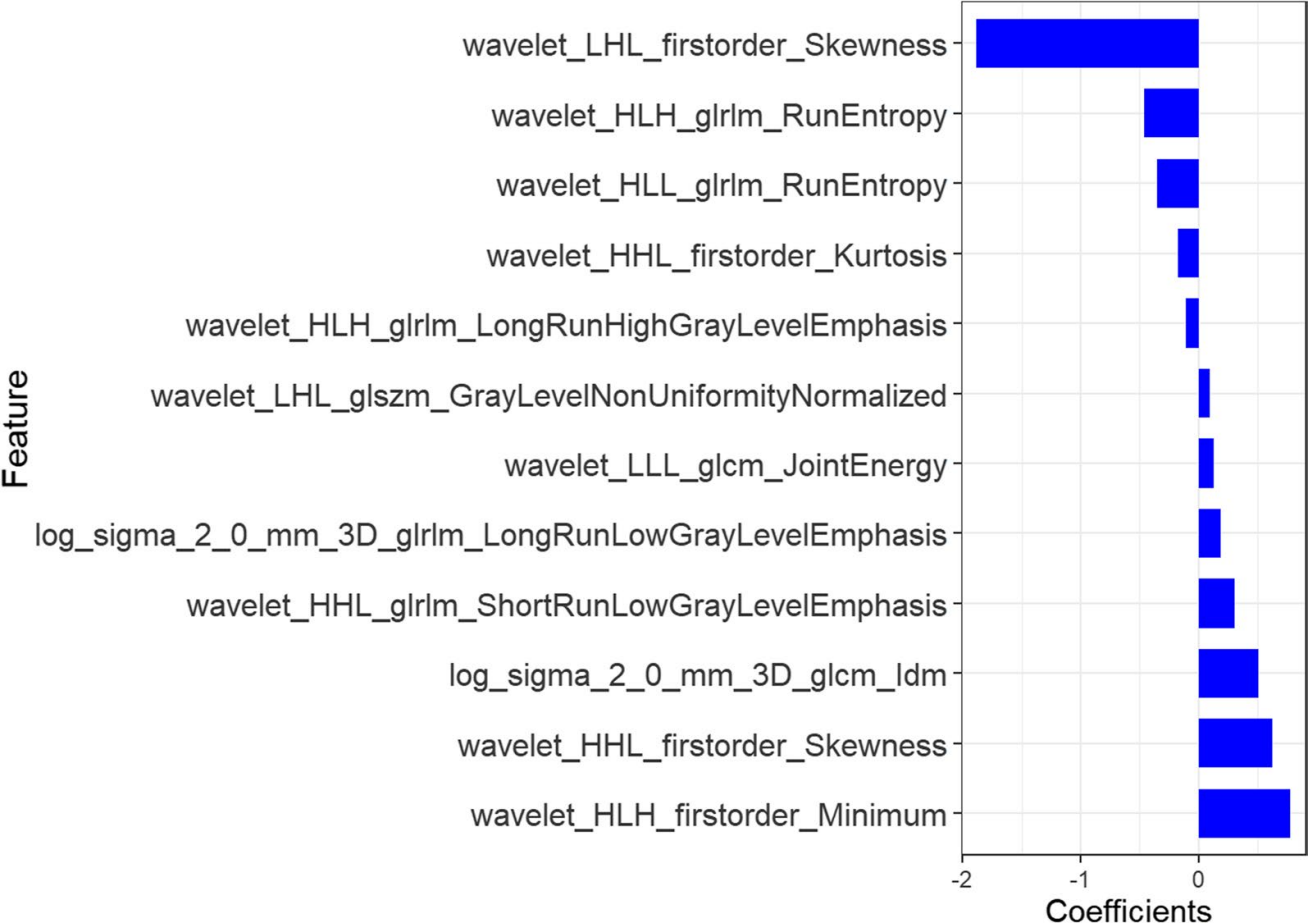
Twelve features obtained after screening and their regression coefficients

After extracting the features, two feature selection methods (mRMR and LASSO) were used to select features. First, mRMR was performed to eliminate redundant and irrelevant features, and 30 features were retained. Next, LASSO was performed to select an optimized subset of features to construct the final model, taking into account the imbalance of the data. Considering the imbalance of the data, a small number of features was oversampled, and 12 features were finally retained (Fig. 2).

### Development and validation of model

**Fig. 3:**
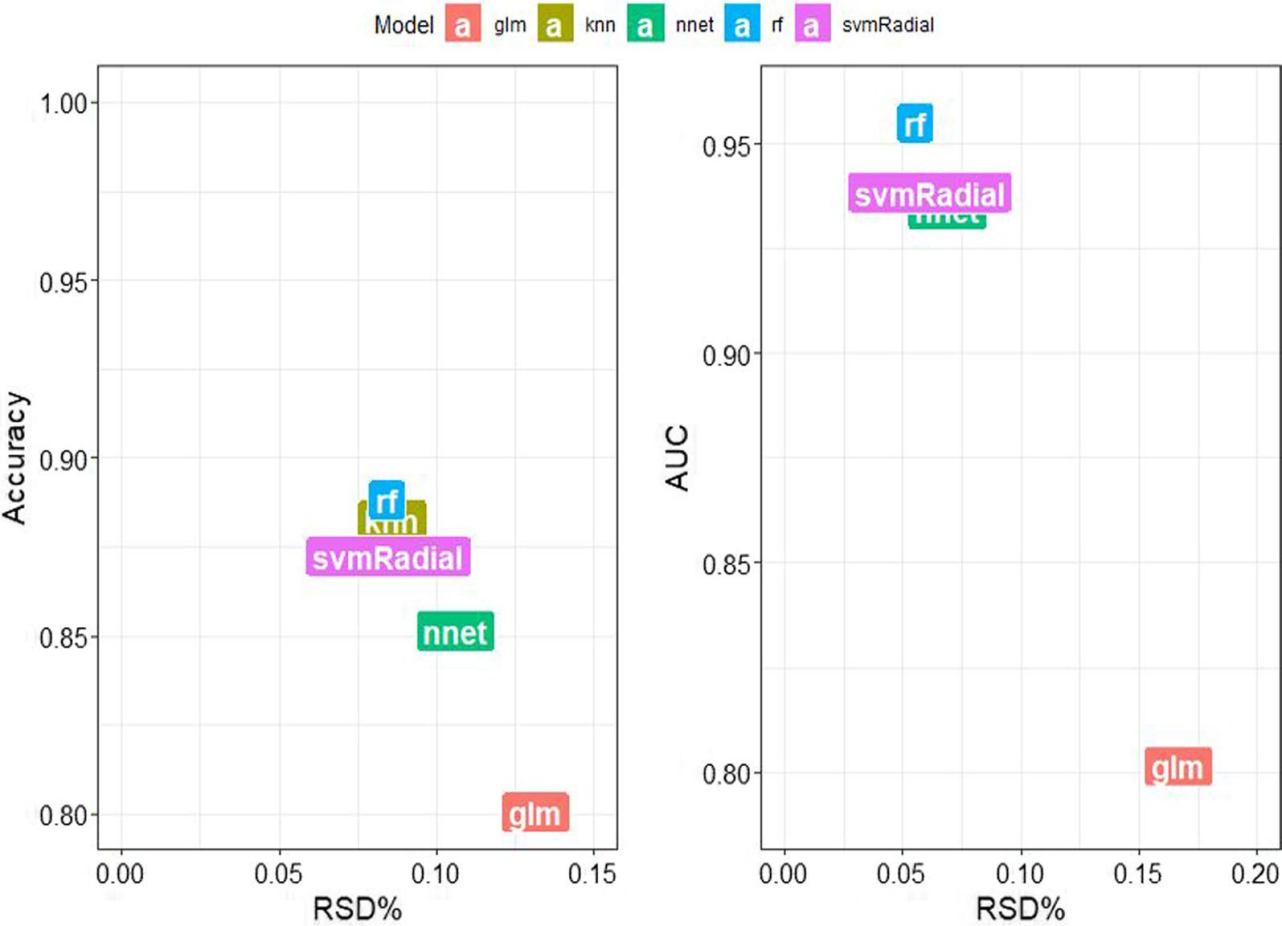
Stability of different models. The ordinate is the average of 100 cross-validations of the AUC and accuracy for each classifier, and the abscissa is the RSD. AUC, area under the curve; RSD, relative standard deviation

**Fig. 4:**
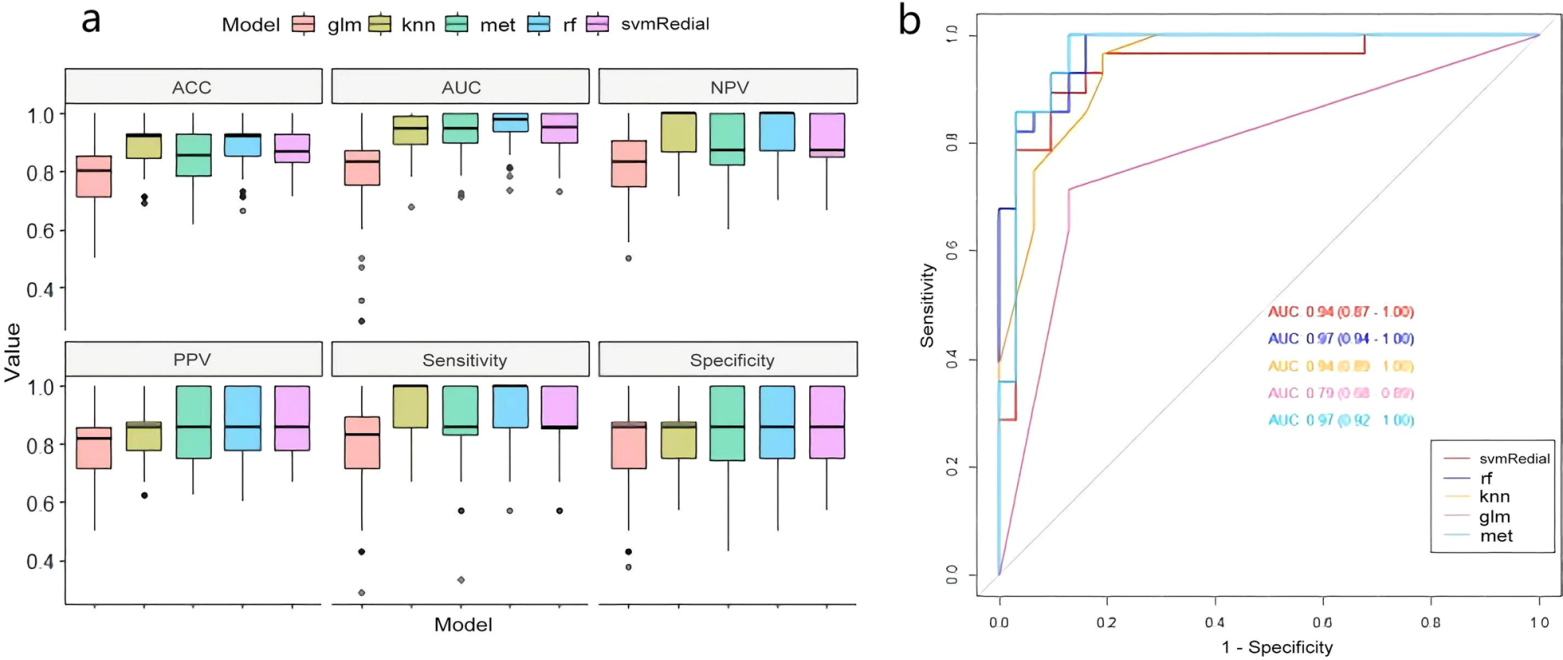
Predictive performance of different models. **a** One hundred cross-validations of the ACC, AUC, NPV, PPV, sensitivity, and specificity of different models. **b** The validation group was used to verify the performance of each model. ACC, accuracy; AUC, area under the curve; NPV, negative predictive value; PPV, positive predictive value

**Fig. 5:**
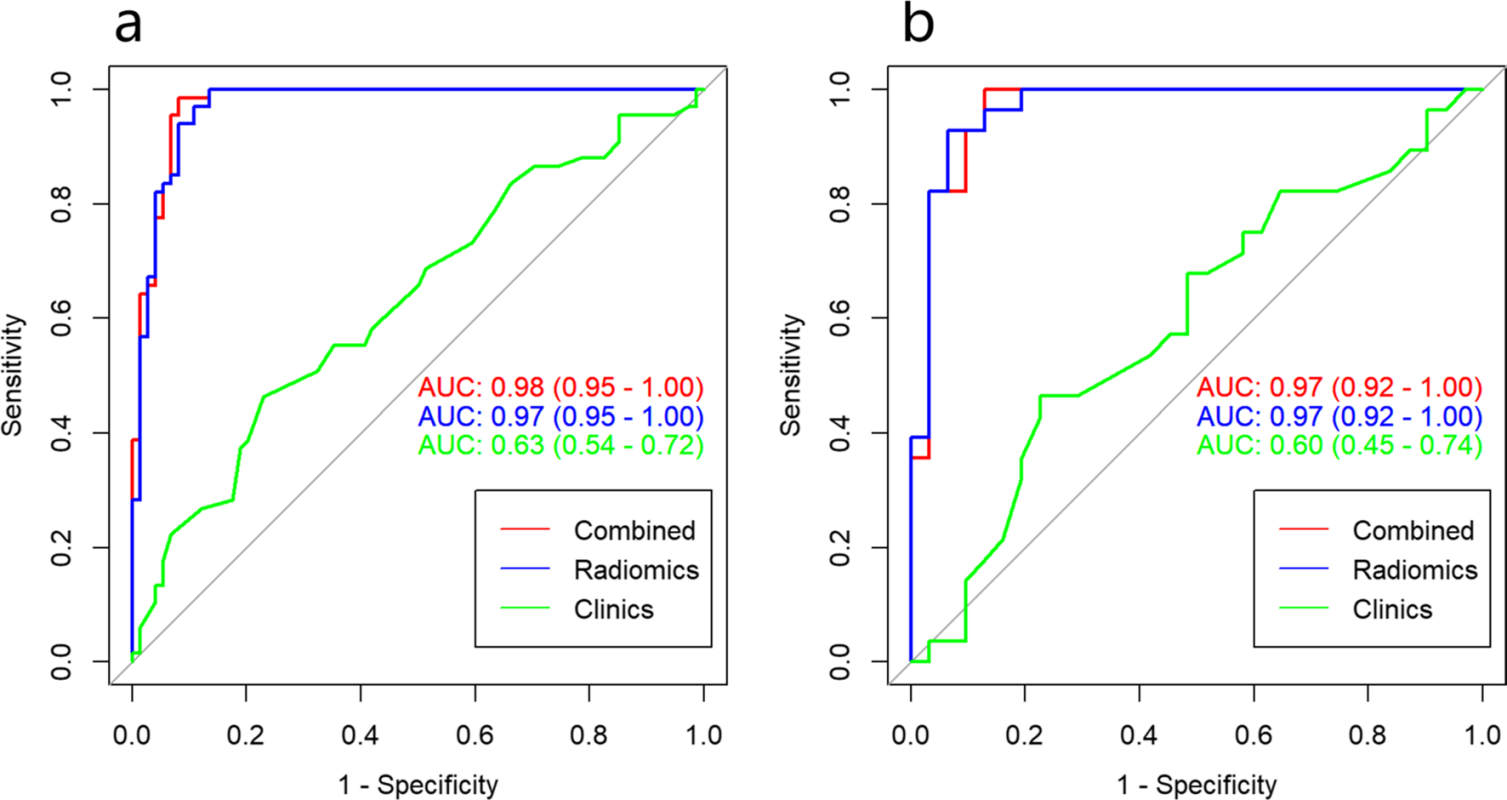
Receiver operating characteristic curves. Comparison of receiver operating characteristic curves to predict calcified plaques. The figure includes **a** training and **b** validation age (green), an radiomics model (blue), and a combined model (red) that combines clinical risk factors and radiomics features. AUC, area under the curve

The 12 radiomics features were used to construct the radiomics model by different machine learning methods. The stability and prediction performance are shown in Figs. 3 and 4. The radiomics model constructed based on the random forest method had the best performance after tenfold cross validation repeated with 10 time, with an AUC of 0.97 (95%CI 0.95–1.00) in the training group and 0.97 (0.92–1.00) in the validation group. In the training group, univariate logistic regression analysis showed that only age (OR 0.953; 95% CI 0.917–0.988; *p* < 0.01) was considered to be an independent predictor (Table 3). The age yielded an AUC of 0.63 (95%CI 0.54–0.72) in the training group and 0.60 (95%CI 0.45–0.74) in the validation group. Finally, multivariate logistic regression analysis was performed to include rad-score and age in the development of a combined radiomics models (Table 3), where rad-score was an independent predictor of calcified plaque (*p* < 0.05).The predictive performance and ROC curves of the model are shown in Table 4 and Fig. 5. The AUC of the combined model were 0.98(95%CI 0.95–1.00), 0.97(95%CI 0.92–1.00), delong test showed that there was no significant difference between the combined model and the radiomics model While the accuracy and specificity were slightly improved (Table 4).

**Table 3:** Univariate and multivariate logistic regression analysis of coronary artery calcified plaques

Variables	Univariate analysis		Multivariate analysis	
	OR (95% CI)	*P*	OR (95% CI)	*P*
Age	0.953 (0.916–0.988)	0.011	1.063 (0.983–1.161)	0.148
Rad-score	–	–	5.627 (3.071–13.463)	0.000

*OR* odd ratio, *CI* confidence interval

**Table 4:** Results of radiomics model, age, and combined radiomics model

	Accuracy	AccuracyLower	AccuracyUpper	Sensitivity	Specificity	Pos.Pred.Value	Neg.Pred.Value
Radiomics							
Train	0.929	0.873	0.965	1.000	0.865	0.870	1.000
Test	0.898	0.792	0.962	0.857	0.935	0.923	0.879
Age							
Train	0.603	0.517	0.684	0.634	0.590	0.388	0.797
Test	0.627	0.491	0.750	0.650	0.615	0.464	0.774
Combined							
Train	0.950	0.900	0.980	0.917	0.986	0.985	0.919
Test	0.932	0.835	0.981	0.875	1.000	1.000	0.871

**Fig. 6:**
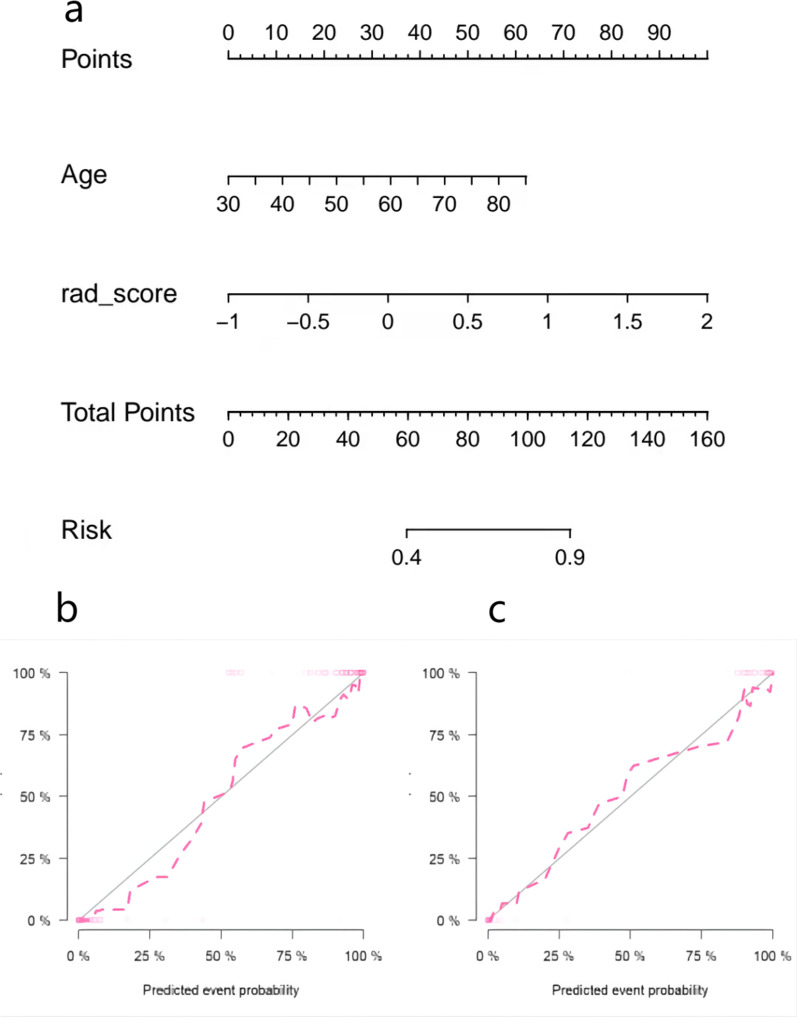
Nomogram of combined radiomics model and consistency curve. **a** Nomogram developed for the main population using a combination of radiomics scores and age factors. The calibration curve was plotted for the **b** training group and **c** validation group. The y-axis represents the actual calcified plaque probability, and the x-axis represents the predicted risk of a calcified plaque. The diagonal line represents the ideal prediction of the ideal model. The pink dotted line indicates the performance of the nomogram; better prediction is achieved as it approaches the diagonal

**Fig. 7:**
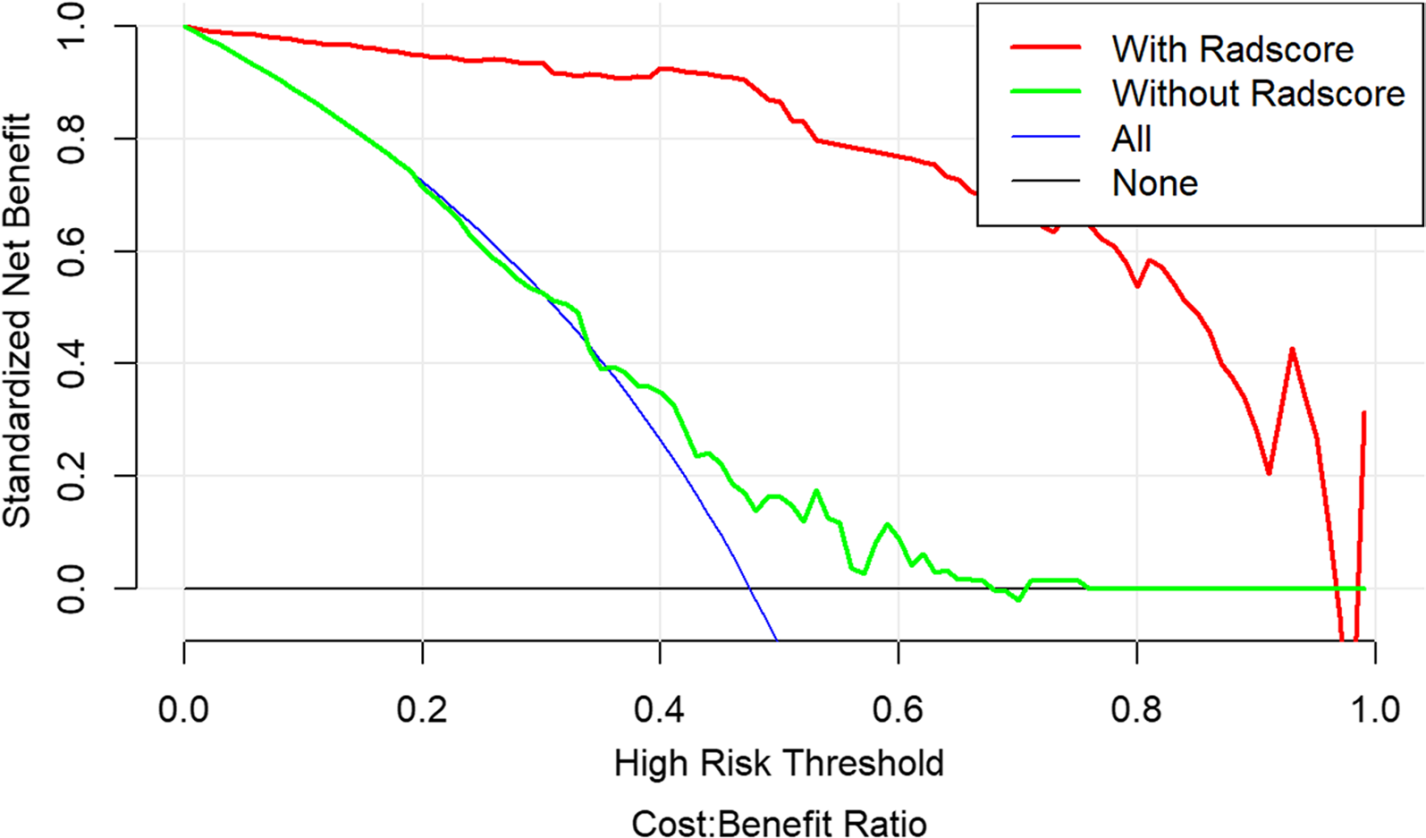
Decision curves of combined model. The x-axis represents the patient’s personal threshold probability (for example, x = 0.6 means that the probability of calcification is 60%), and the y-axis represents the net benefit. The green line represents the clinical factor model. The red line represents the combined radiomics model. The blue line represents the hypothesis that all patients have calcified plaques. The thin black line indicates the assumption that no patient has plaques. The net benefit is calculated by subtracting the proportion of all false-positive patients from the proportion of true positives, and then weighted according to the relative harm of previous treatment and the negative consequences of unnecessary treatment. The decision curve shows that the performance of the combined radiomics model is much higher than the remaining three items

The calibration curve was used to evaluate the consistency between the predicted probability and the actual observed value. The Hosmer–Lemeshow test was used to test the difference between these values. The training group *p* = 0.535, the validation group *p* = 0.383, and *p* > 0.05 indicated good agreement between the predicted probability and the actual observed value (Fig. 6). Using the decision analysis curve indicates that the combined model has a high clinical utility. These results indicate that the combination of radiomics features and clinical risk factors provides more effective information for assessing whether a plaque is calcified and therefore has higher clinical application value (Fig. 7).

## Discussion

Research has shown that the progression of coronary atherosclerotic plaques is related to the calcification component. This starts with intimal microcalcifications in the early stages of atherosclerotic plaque formation and progresses to fibrous atherosclerotic punctate and fragment calcifications, which are relatively unstable and lead to a greater risk of plaque rupture. On CT, they tend to show low-attenuation non-calcified plaque, the napkin ring sign, positive remodeling, and spotty calcification. In later stages, plaque calcification gradually turns into lamellar calcification and calcified nodules, which are relatively stable deposits of calcium ions in tissue cells after the arterial injury has recovered. They appear as patchy dense shadows with a higher density than the lumen on CCTA, with CT values of > 1000 HU [19, 20]. Boussel et al. [21] demonstrated that quantitative energy-spectral CT parameters were effective in identifying calcified and lipid-rich plaques in coronary arteries by analyzing differences in single-energy CT values. However, the limitations of their study were the small sample size of 23 cases and the ex vivo experimental design. In recent years, research has shown that adipose tissue is a key regulator of healthy cardiac metabolism and that PVAT plays a key role in vascular system homeostasis and atherogenesis by regulating the local microenvironment through the release of bioactive adipokines, gases, and other lipid messengers outside the vasculature [22–24].

In this study, we proposed a model that combines clinical risk factors and radiomics features of PVAT to predict plaque calcification. First, PVAT radiomics analysis can significantly distinguish between non-calcified and calcified plaques. The AUC in the training group was 0.97, and that in the validation group was 0.97. Second, univariate logistic regression showed that the clinical factor closely associated with differentiating calcified plaque was age, suggesting that clinical attention should be paid to coronary artery examination in elderly patients to keep abreast of lesion plaque progression. However, the predictive performance for calcified plaque was limited, with a validation group AUC of 0.60. with a validation group AUC of 0.60. Therefore, we developed a combined model to test whether radiomics features and age are complementary. The nomogram based on the integrated model exhibited the most ideal performance in the training and validation group, with an AUC of 0.98 and 0.97, respectively.

In clinical factors, only age in the training group was relevant in this study. Hypertension, diabetes, and dyslipidemia, as clinical factors associated with cardiovascular risk, were not relevant for identifying calcified plaques in this experiment. The improvement of the combined radiomics model by clinical risk factors was not substantially great. This may have been caused by the limited sample size, and a sufficiently large sample may produce better statistics.

This study had several limitations. First, this study may have had selection bias because of its retrospective nature. Prospective, multicenter, and large-sample randomized controlled research is therefore necessary. Second, only calcified and non-calcified plaques were analyzed in this study, and mixed plaques were not included for comparison. Third, our results only support the detection of PVAT characteristics in symptomatic patients; the clinical value of our findings in asymptomatic patients remains unclear. Fourth, the degree of coronary stenosis is an important indicator but is susceptible to calcified plaque X-ray sclerosis artifacts, which are difficult to accurately assess and were not included in the analysis. Finally, the plaques sampled in this study were not pathologically confirmed, and it was not possible to accurately group the plaques.

## Conclusion

We verified that age and radiomics features had good predictive ability for calcified plaques. We propose a noninvasive method that can assist the clinical diagnosis and decision-making for patients with plaques.

## Data Availability

The data that support the findings of this study are available from the corresponding author. However, restrictions apply to the availability of these data, which were used under license for the current study and are therefore not publicly available. However, data are available from the authors upon reasonable request and with permission of the corresponding author.

## Abbreviations

AUC: Area under the curve
CCTA: Coronary CT angiography
CI: Confidence interval
CT: Computed tomography
GLCM: Gray-level co-occurrence matrix
GLSZM: Gray-level size zone matrix
GLRLM: Gray-level run-length matrix
LASSO: Least absolute shrinkage and selection operator
mRMR: Maximum correlation minimum redundancy
PVAT: Perivascular adipose tissue
ROC: Receiver operating characteristic curve
